# Supplementary material for: Manual Therapy Facilitates Homeostatic Adaptation to Bone Microstructural Declines Induced by a Rat Model of Repetitive Forceful Task
Source: Int J Mol Sci. 2022 Jun 13;23(12):6586. doi: 10.3390/ijms23126586 (PMC9223642; doi:10.3390/ijms23126586)
Supplement: Supplementary file 1 [file ijms-23-06586-s001.zip › ijms-1743250-supplementary.pdf]

International Journal of
Molecular Sciences

MDPI

Supplementary Material

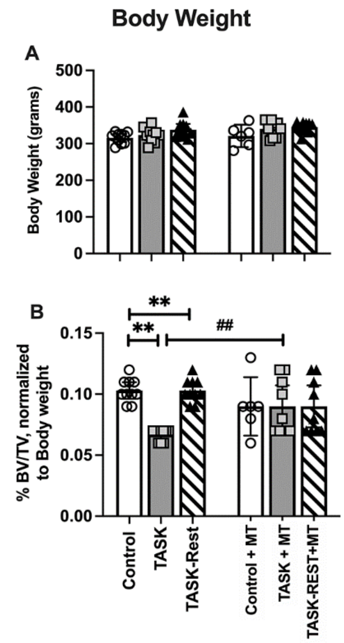

Figure S1. Body weight of each group at the time of tissue collection. (**A**) Body weight in last week before tissue collection. (**B**) Percent BV/TV after normalization to body weight.or 6 weeks after task cessation (TASK-R); control rats that received manual therapy for 12 weeks (Control+MT); TASK rats that simultaneously received manual therapy for 12 weeks (TASK+MT); TASK rats that rested for 6 weeks after task cessation while receiving manual therapy treatment of their upper limbs three times per week (TASK-MTR). ##: $p < 0.01$, compared to the TASK group. **: $p < 0.01$, compared between groups as shown. n.s. = not significant.
